# Point cloud dataset of mature pecan trees (*Carya illinoinensis*) obtained with a low-cost mobile terrestrial LiDAR

**DOI:** 10.1016/j.dib.2026.113076

**Published:** 2026-07-13

**Authors:** Raúl Porras, Gilberto Rivera, Vicente García, Rogelio Florencia, Patricia Sánchez-Solís

**Affiliations:** Departamento de Ingeniería Eléctrica y Computación, Universidad Autónoma de Ciudad Juárez, Av. del Charro y Henry Dunant, Ciudad Juárez, 32310, Chihuahua, Mexico

**Keywords:** LiDAR, 3D point clouds, Mobile terrestrial laser scanning, Point cloud denoising, Precision agriculture, Plant phenotyping, Tree architecture

## Abstract

This article describes a comprehensive point cloud dataset representing the 3D structures of 33 mature pecan trees (*Carya illinoinensis*, cv. 'Western'), which was acquired *in situ* within commercial orchards in Chihuahua, Mexico. The data were obtained using a low-cost mobile terrestrial laser scanner, consisting of an iPad Pro (11-inch, M1 processor). A pedestrian-based kinematic scanning protocol was applied, based on the EveryPoint application, which leverages the device’s integrated LiDAR sensor and its 12-megapixel Wide camera. The dataset contains point clouds at several stages of processing, ranging from raw acquisitions containing inherent sensor noise and positional artifacts (the “noise dome”) to definitively filtered, ready-to-use spatial geometries. Data processing was carried out using programmatic preprocessing, which involved geometric filtering based on local volume density and Gaussian curvature thresholds, in order to systematically remove outliers near the woody branching architecture. The dataset is available in standard .ply and .las formats, and is accompanied by *in situ* photographic reference images. It may be used to develop and benchmark denoising methods for low-cost LiDAR data, or to support non-destructive phenotyping and pruning-related decision support applications in pecan orchards.

Specifications Table

**Table:** 

Subject	Biology
Specific subject area	Agricultural sciences; Agronomy and crop science; Agricultural sciences with computational support
Type of data	3D point cloud datasets (.ply, .las); photographic reference images (.jpg, .png)
Data collection	Structural data on 33 mature pecan trees (∼12 years old) were acquired *in situ* using a low-cost MTLS configuration (iPad Pro 11-inch, M1, solid-state LiDAR). Data were acquired with the EveryPoint application, using the device’s integrated LiDAR sensor and a vision-aided SLAM framework. A pedestrian-based kinematic scanning protocol was followed, with a speed of approximately 3 km/h along a 360° circular trajectory with a ∼3 m radial offset around each tree. For optimal capture of the upper canopy, the device was mounted on a 3 m extension pole. Raw point clouds were pre-processed and filtered using geometric descriptors to mitigate spatial noise and isolate the definitive woody structure.
Data source location	City: Ciudad Delicias, Chihuahua; Country: Mexico
Data accessibility	Repository name: Zenodo DOI: https://doi.org/10.5281/zenodo.21055596 Direct URL to data: https://zenodo.org/records/21055596
Related research article	None

## 1 Value of the Data

• This dataset contains 3D point cloud representations of the architecture of mature pecan trees, and was created in response to the limited availability of crop-specific LiDAR datasets beyond forest ecosystems and high-density fruit trees such as apple and citrus.
• Raw and sequentially filtered point clouds were acquired using a low-cost mobile terrestrial laser scanner (MTLS); both types are included in the dataset to enable the evaluation of low-cost sensing technologies for high-throughput plant phenotyping.
• Researchers in computer vision, point cloud processing, and computational geometry can use the multi-stage data (raw, filtered, and processed point clouds) to develop, train, and benchmark denoising and outlier removal algorithms for complex woody structures.
• Researchers and practitioners in agronomics and precision agriculture can use these processed 3D architectures to model pruning strategies under structural constraints (e.g., the removal of a maximum of 30% woody volume and the retention of 40–60 cm fruiting shoots), thereby supporting non-destructive orchard management.
• Point clouds at intermediate stages of processing are included to facilitate studies of reproducibility and comparative evaluations of filtering pipelines based on geometric descriptors such as local density and Gaussian curvature.

## 2 Background

To optimize the longevity and yield of pecan trees [[1], [2], [3]], structural pruning interventions are often applied. For effective management, strict constraints must be met, such as limiting the removal of woody material to 30% by volume [[4], [5], [6], [7]]. However, conventional pruning methods are largely empirical, and a transition to quantitative, data-driven decision support models will require high-fidelity 3D phenotyping. Although specialized terrestrial LiDAR is financially prohibitive, low-cost MTLSs offer a scalable, affordable alternative.

The LiDAR datasets that are currently available to the public typically feature forest ecosystems or simpler, high-density crops [[8], [9], [10]]. There is a lack of scanned 3D data for complex pecan architectures, which impedes the benchmarking of point cloud processing algorithms.

To address this gap, this article presents a 3D dataset of woody architectures for mature pecan trees, which was acquired using a low-cost MTLS. This dataset represents a benchmark for the training and optimization of denoising and outlier-removal algorithms tailored to noisy sensor data. Ultimately, it will enable the development of accessible, data-driven decision-support tools for precise orchard management and non-destructive structural analysis.

## 3 Data Description

Point cloud datasets were acquired *in situ* within a commercial orchard located in the municipality of Delicias, Chihuahua, northern Mexico. This agricultural site is characterized by predominantly clay soil and is managed using a traditional flood irrigation system. A cohort of 33 individual fruit-bearing pecan trees, all approximately 12 years old, was scanned. The dimensions of the orchard are 12×12 m, and the trees are structurally managed under a central leader pruning system.

Data acquisition was conducted only during the phenological stage of winter dormancy (leafless condition), since structural pruning remains a viable and critical annual agronomic practice for actively producing trees of this age. The individual trees were selected because they were particularly suitable for an MTLS dataset; the moderate crown volume of each tree, its defoliated state, and the absence of excessively vigorous, hyper-dense branching significantly mitigated laser occlusion, enabling high-fidelity digitization of the woody architecture.

**Fig. 1:**
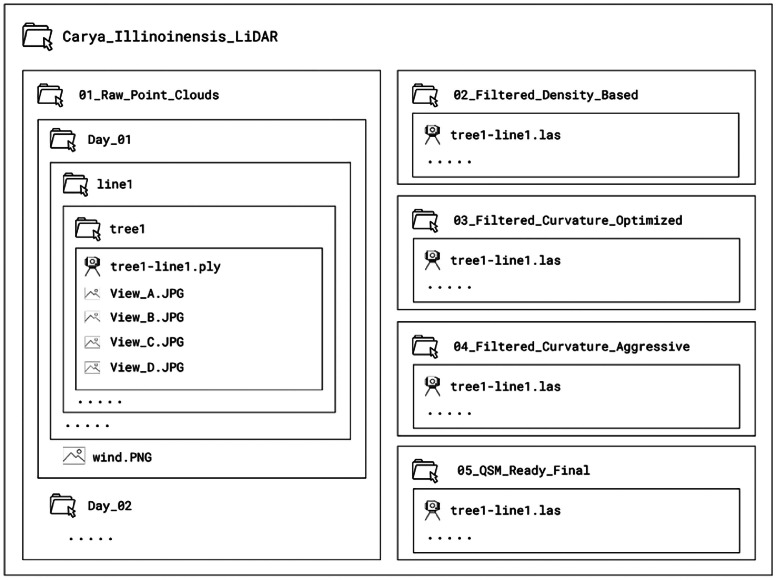
Directory structure and organization of the dataset.

The dataset is made available as a single compressed archive called Carya_Illinoinensis_LiDAR.zip. The extracted files are organized into five primary directories representing the data processing pipeline, as illustrated in Fig. 1. The foundational directory, 01_Raw_Point_Clouds, is subdivided into five folders corresponding to the days on which data were acquired (e.g., Day_01). Each of these folders includes a meteorological reference file (e.g., wind.PNG) that summarizes the environmental conditions during the scanning sessions. Each folder also contains subdirectories denoting the rows of the orchard (e.g., line1), which in turn contain individual folders for each scanned tree (e.g., tree1). Each folder representing a tree contains a raw 3D point cloud, using a standardized nomenclature: tree[X]-line[Y].ply, where [X] indicates the individual's position within the row, and [Y] designates the row number within the orchard block (e.g., tree1-line1.ply). Accompanying each point cloud are four *in situ* JPG photographs of the target pecan tree. These images provide a multi-view visual reference that can support qualitative validation of the point clouds. They are captured from four distinct peripheral perspectives, and are named View_A.JPG through View_D.JPG.

The other directories contain computationally filtered instances representing different denoising methodologies. The folder named 02_Filtered_Density_Based contains point clouds processed with CloudCompare's Geometric Features module, in which a local volume density strategy based on the number of nearest neighbors is employed. The folders named 03_Filtered_Curvature_Optimized and 04_Filtered_Curvature_Aggressive contain datasets filtered using Gaussian curvature metrics. These two sets differ in the stringency of the scalar thresholds that were applied, representing a contrast between a conservative structural retention strategy (optimized) with a more aggressive noise reduction process (aggressive).

The final directory, 05_QSM_Ready_Final, contains the filtered point clouds. For these instances, expert-guided manual outlier removal was applied following the curvature filtering, in order to eliminate residual artifacts from the raw data. Notably, although the nomenclature of the processed files in all of these subdirectories strictly adheres to the previously established spatial nomenclature (e.g., tree1-line1), the file format is changed from .ply to .las to maximize interoperability with standard dasonomic and geospatial modeling software.

Quantitative structure models (QSMs) are geometric reconstructions that represent the architecture of a tree as a collection of interconnected cylinders, thus enabling structural attributes such as the branch length, diameter, and woody volume to be estimated [11]. However, a QSM reconstruction is highly sensitive to noise and outliers in the input point clouds. For this reason, the final filtered dataset is provided in a QSM-ready format, with residual artifacts removed, to ensure topological consistency and reliable model fitting. In particular, the curation process enforces branch-level continuity and removes interstitial noise that would otherwise disrupt the cylinder fitting and reconstruction of branching topology in QSM algorithms.

In terms of data volume, the uncompressed repository requires approximately 8.9 GB of storage capacity. The internal data structure of the .las files is exclusively based on the 3D spatial geometry (XYZ coordinates) of the point clouds. Conversely, the .ply files encapsulate both the spatial coordinates and their corresponding RGB (red, green, blue) colorimetric attributes acquired by the sensor.

A comprehensive inventory of the *in situ* structural measurements for each of the 33 fully processed pecan trees is presented in Table 1. This table provides the physical context and supplementary metadata for the scanned orchard, with details of the total height, crown length, crown diameter, and diameter at breast height (DBH) for each individual tree. To ensure methodological consistency with standard forestry practices, DBH was defined as the trunk diameter measured at 1.30 m above ground level, while the crown length was taken as the vertical distance from the lowest primary branch to the highest apical point.

**Table 1:** *In situ* structural ground truth measurements for 33 mature pecan trees.

Raw file	Total height (m)	Crown length (m)	Crown diameter (m)	DBH (cm)
tree1-line1.ply	9.47	7.43	8.4	64.6
tree1-line2.ply	4.91	3.31	3.36	28.2
tree1-line4.ply	8.66	6.56	7.98	69.2
tree1-line5.ply	7.62	5.25	5.27	49
tree1-line6.ply	8.40	6.65	7.95	55
tree1-line7.ply	11.66	9.38	8.05	68
tree2-line1.ply	9.09	7.25	8.74	66.2
tree2-line2.ply	7.30	5.70	5.39	44.2
tree2-line3.ply	6.65	4.75	4.13	37.2
tree2-line4.ply	9.98	7.93	7.65	68.6
tree2-line6.ply	7.81	5.86	6.95	54
tree2-line7.ply	5.35	3.61	1.67	25
tree3-line1.ply	10.03	8.52	8.77	69.2
tree3-line3.ply	7.83	6.47	6.42	52.8
tree3-line5.ply	8.38	6.44	7.87	57.8
tree3-line6.ply	8.01	6.12	8.34	58.2
tree3-line7.ply	7.10	1.57	4.37	41
tree4-line5.ply	7.18	5.68	5.48	41.6
tree4-line6.ply	5.82	4.42	3.93	33.2
tree4-line7.ply	7.17	5.85	6.73	56.8
tree5-line4.ply	6.83	4.83	5.30	54
tree5-line5.ply	7.16	6.02	6.65	50
tree6-line4.ply	7.95	6.38	7.40	55
tree6-line5.ply	9.90	8.24	7.95	54
tree6-line6.ply	10	7.98	8.44	68.8
tree6-line7.ply	8.28	7.28	6.45	55
tree7-line5.ply	8.38	6.24	7.17	52.4
tree7-line6.ply	9.84	8.11	6.85	60.2
tree8-line6.ply	7.46	5.77	6.41	47.2
tree8-line7.ply	10.64	8.96	8.76	66.6
tree8-line8.ply	8.56	6.80	7.82	59.8
tree9-line7.ply	10.57	9.22	8.10	62.2
tree9-line8.ply	8.48	6.50	6.04	50.6

## 4 Experimental Design, Materials and Methods

Structural data were acquired using a low-cost MTLS based on the solid-state LiDAR sensor integrated into an iPad Pro (11-inch, M1 processor). Point clouds were acquired via the EveryPoint application, which uses the device’s integrated direct time-of-flight (dToF) solid-state LiDAR sensor and its 12-megapixel Wide camera (f/1.8 aperture) to capture both the 3D spatial geometry and true color (RGB) attributes simultaneously, via a vision-aided simultaneous localization and mapping (SLAM) framework.

**Fig. 2:**
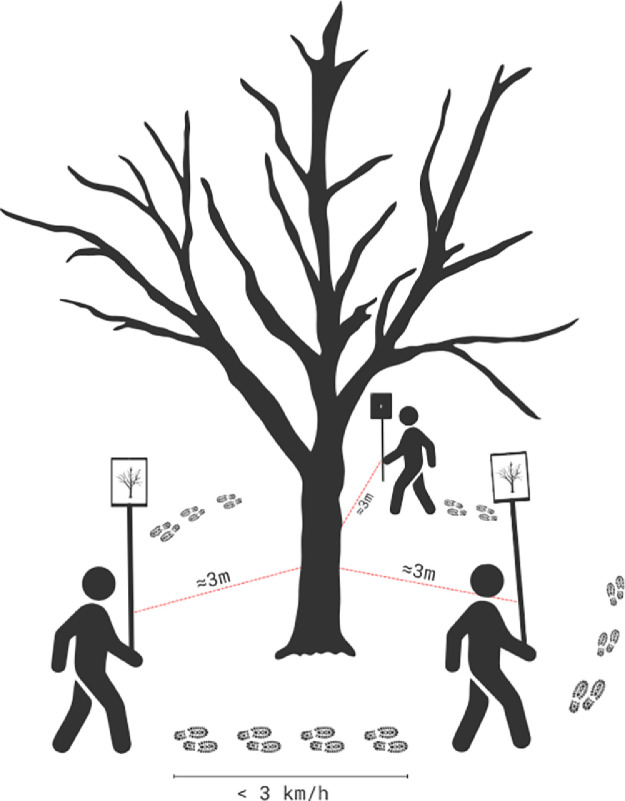
Diagram illustrating the scanning strategy.

Data acquisition followed a pedestrian-based kinematic scanning protocol, as shown in Fig. 2. The operator maintained a sub-walking speed (approximately 3 km/h) while following a circular trajectory with a radial offset of ∼3 m around each target pecan tree, providing 360° coverage. To capture the upper woody crown architecture within the sensor's operational range (approximately 5 m), the device was mounted on a 3 m tripod extension.

**Fig. 3:**
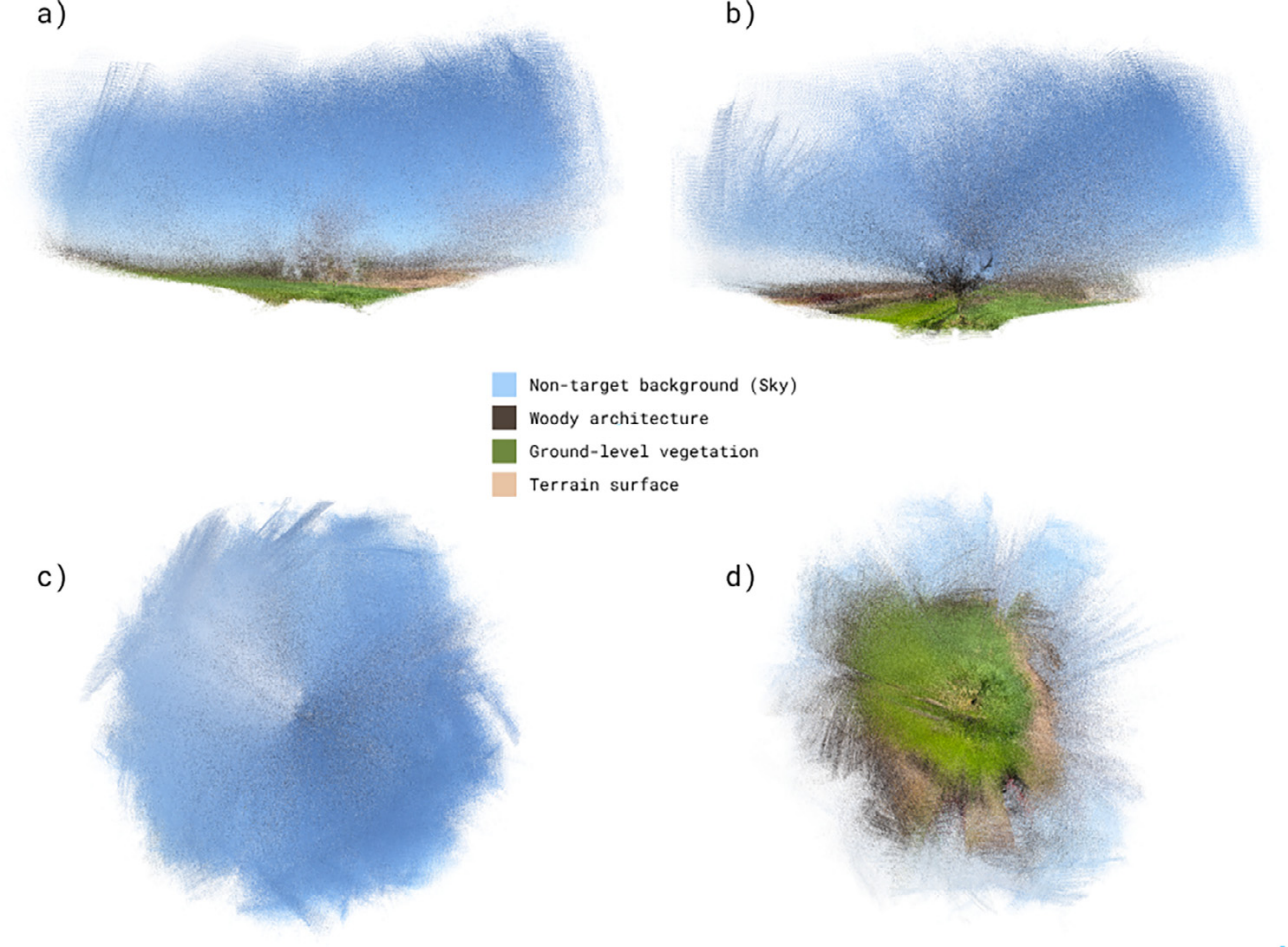
Multi-perspective, true-color 3D LiDAR point clouds for pecan trees: (a) full cloud, lateral perspective; (b) axial vertical cross-section; (c) nadir (top-down) perspective; and (d) zenithal (bottom-up) perspective. Point colors are derived from in situ RGB imagery captured by the sensor at the same time as the architecture data, and represent the scene components (sky, woody architecture, ground-level vegetation, and terrain surface) as shown in the legend. No artificial classification-based coloring is employed.

The raw point clouds contain spatial artifacts that are associated with sensor noise and positional drift. As illustrated in Fig. 3, these effects generate a dense hemispherical structure, often referred to as a “noise dome,” surrounding the tree's geometry. Visual identification of these artifacts is facilitated by RGB values, which were recorded simultaneously with the spatial coordinates using the device's integrated optical camera.

### 4.1 Data Processing

Prior to applying the curation procedures for curvature-, density-, and QSM-ready instances, each raw point cloud underwent an initial preprocessing step, which was implemented using a custom Python script. Three filters were applied sequentially to isolate the target pecan tree from the surrounding macroscopic noise artifact (the “noise dome”). A colorimetric threshold was first used to isolate wood-toned structural elements via logical RGB masks (80≤r≤160,40≤g≤100,20≤b≤70,subject to specific inter-channel constraints such as (r+g+b)<300) while explicitly filtering out gray/black background noise (where max⁡(|r−g|,|r−b|,|g−b|≤20) and mean RGB intensity ≤100). Following this, a Cartesian spatial bounding box was imposed to eliminate ground and distant artifacts, using empirically determined coordinate limits (in meters): x∈[−3.37,2.77], y∈[−1.00,6.15], and z∈[−1.60,3.89]. Finally, a standard radius outlier removal (ROR) algorithm was applied, which was parameterized to require at least 30 neighboring points within a 0.07m search radius.

**Fig. 4:**
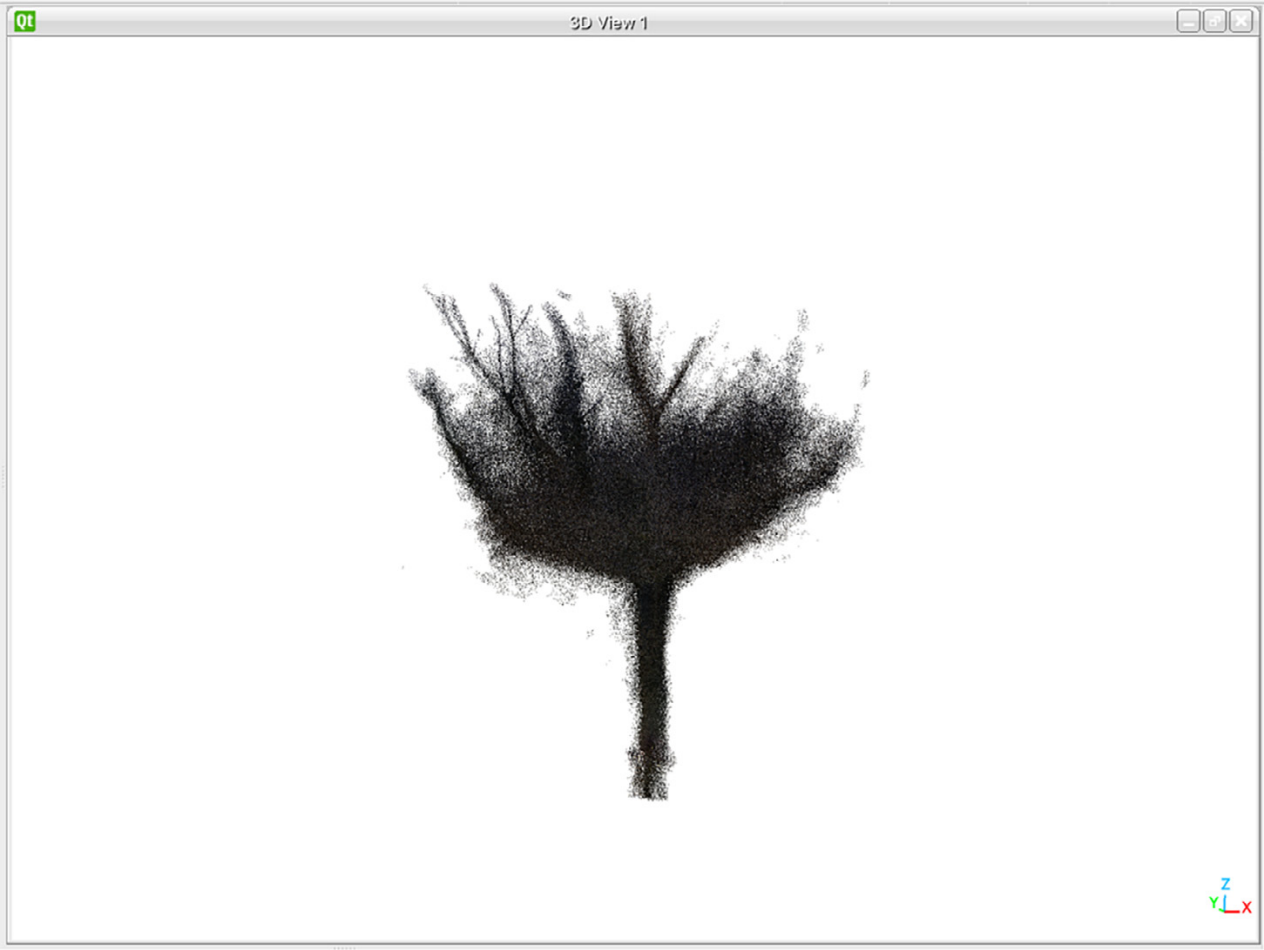
Point cloud after Python-based preprocessing.

Although the tree can be separated from this large-scale artifact using spatial bounding, residual noise remains near the woody structure. Immediately after this Python-based preprocessing step, the point cloud still contains dense noise artifacts that are entangled with the branching framework, as illustrated in Fig. 4.

These preprocessed point clouds were then processed using CloudCompare's *Compute Geometric Features* (Version 2.13.2) [12]. Local neighborhood density and Gaussian curvature descriptors were computed as scalar fields and subsequently filtered using the *Filter by Value* algorithm to remove proximal outliers.

**Fig. 5:**
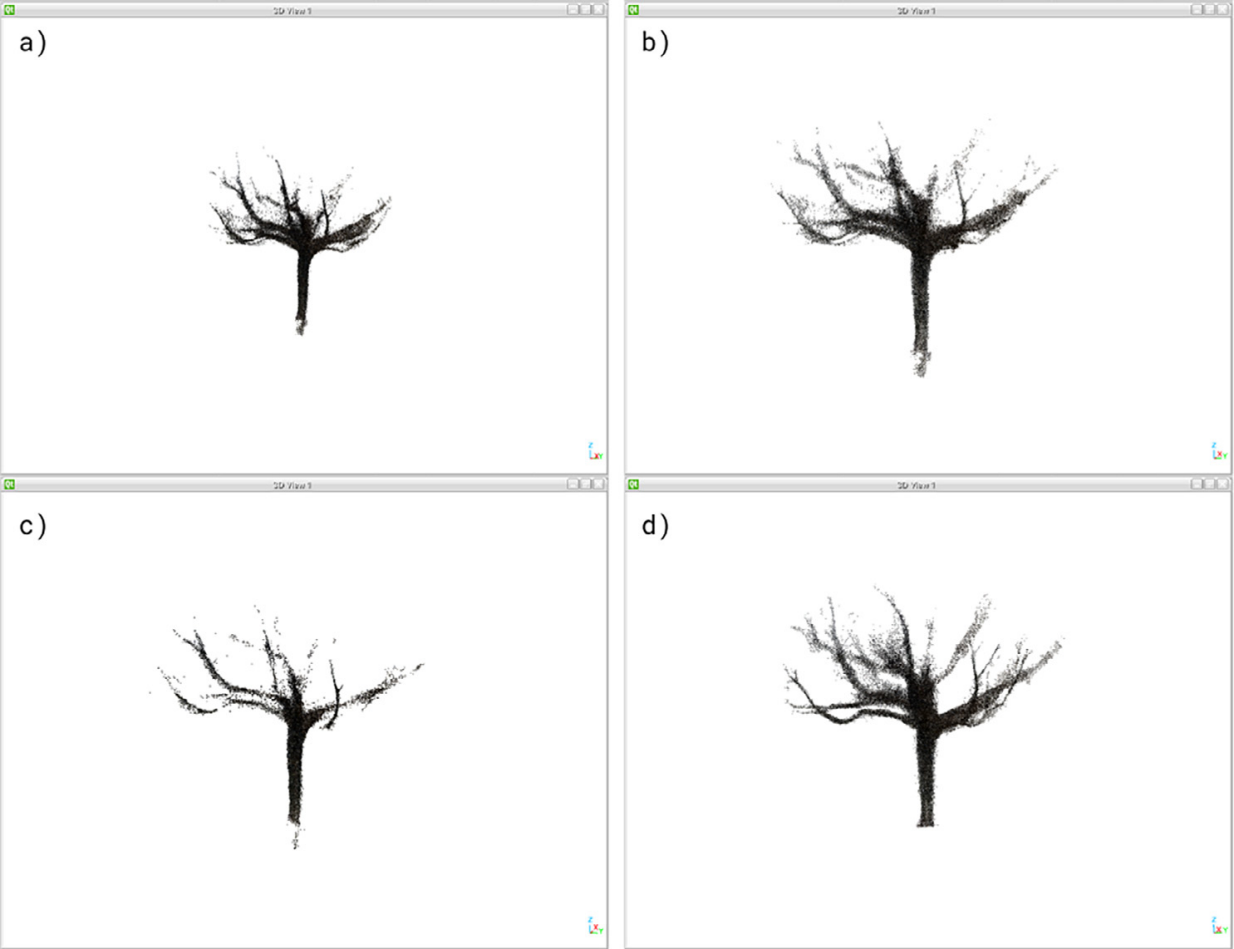
Point clouds at different stages of the filtering process, showing the results after (a) spherical neighborhood radius filtering; (b) Gaussian curvature descriptor filtering with r=0.025; (c) Gaussian curvature descriptor filtering with r=0.01; (d) optimized curvature filtering.

For the datasets in the 02_Filtered_Density_Based directory, a spherical neighborhood radius of r=0.01 was used, and a maximum threshold of three was applied to the density descriptor (Fig. 5a). The 03_Filtered_Curvature_Optimized directory contains datasets processed using the Gaussian curvature descriptor with a radius of r=0.025 and a maximum threshold of 40 (Fig. 5b). In the 04_Filtered_Curvature_Aggressive directory, the Gaussian curvature descriptor was applied with a radius of r=0.01, using the default threshold determined by the software (Fig. 5c). The preprocessing scripts used in this study are available in the Zenodo repository [13], including parameter configurations and execution instructions.

In the last step, the 05_QSM_Ready_Final dataset was generated by applying optimized curvature filtering (radius r=0.025, threshold 40) and then a systematic, multi-perspective manual refinement protocol (Fig. 5d). Since automated filtering isolated the macro-structure but left artifacts that would prevent the accurate generation of QSMs, CloudCompare's manual segmentation tool was applied to execute a final visual-spatial curation. This expert-guided phase was governed by three strict morphological criteria:

• Excision of residual proximal noise intimately entangled with the branches;
• Isolation and removal of interstitial artifacts (points suspended between distinct branching axes), which were continuously verified through 3D rotational inspection to ensure they did not belong to occluded branches;
• Geometric thinning of the woody components to accurately delineate the true topological skeleton of the tree.

Manual curation followed this standardized protocol, which was applied consistently to all instances. Although these criteria are inherently operator-dependent, they were strictly applied and iteratively reviewed through multi-view inspection.

Since the data were produced in QSM-ready format, this dataset can be used directly for structural modeling workflows without the need for extensive additional preprocessing. To facilitate reuse, the .las files can be loaded directly from the 05_QSM_Ready_Final directory into standard point cloud processing tools (e.g., CloudCompare and MATLAB-based QSM frameworks). The multi-stage structure also allows users to benchmark denoising pipelines by comparing raw, intermediate, and filtered datasets.

As a quantitative summary of the efficacy of this filtering pipeline, a comprehensive inventory of the instances and the spatial quality metrics for the point clouds are presented in Table 2. This table illustrates the refinement of the datasets, from the initial raw acquisitions to the final QSM-ready woody architectures. To establish a rigorous baseline for the spatial resolution, the mean point density was calculated using a 0.10 m volumetric spherical neighborhood for the raw point clouds. The structural fidelity of the post-processed data was evaluated at the same time by measuring the filtered noise, which represents the local surface roughness of the definitive geometry. This roughness metric was computed based on a 0.03 m kernel radius, in order to capture sub-centimeter sensor artifacts, and demonstrates the geometric clarity and noise reduction achieved by the proposed filtering workflow.

**Table 2:** Inventory and spatial quality metrics of the point clouds, showing the progression from raw acquisition to the final filtered woody architecture.

Raw file	Raw point count	Filtered point count	Mean raw density $\left(\mathrm{pts}\;/\;m^{3}\right)$	Mean filtered density $\left(\mathrm{pts}\;/\;m^{3}\right)$	Mean roughness (cm)
tree1-line1.ply	13,355,200	48,189	58,952	36,336	0.53
tree1-line2.ply	13,409,600	30,835	38,747	33,850	0.54
tree1-line4.ply	14,693,440	70,138	139,854	39,278	0.51
tree1-line5.ply	14,394,240	60,466	146,235	31,089	0.55
tree1-line6.ply	25,043,040	76,600	158,727	36,184	0.54
tree1-line7.ply	23,929,200	123,834	256,510	38,151	0.52
tree2-line1.ply	15,090,560	69,925	86,984	38,837	0.50
tree2-line2.ply	13,186,560	51,864	60,282	34,970	0.53
tree2-line3.ply	13,757,760	83,289	152,861	36,637	0.53
tree2-line4.ply	14,263,680	72,566	148,956	33,017	0.54
tree2-line6.ply	13,371,520	142,156	123,180	28,451	0.58
tree2-line7.ply	22,338,000	83,447	98,515	38,585	0.51
tree3-line1.ply	17,440,640	103,915	77,632	33,134	0.54
tree3-line3.ply	13,034,240	76,540	166,469	34,698	0.53
tree3-line5.ply	12,892,800	70,125	98,045	31,405	0.56
tree3-line6.ply	25,655,040	110,872	236,140	34,506	0.63
tree3-line7.ply	24,859,440	67,527	120,232	38,285	0.53
tree4-line5.ply	19,498,320	78,239	77,616	35,982	0.55
tree4-line6.ply	16,169,040	55,824	148,693	39,967	0.54
tree4-line7.ply	26,854,560	109,282	225,527	39,600	0.53
tree5-line4.ply	12,218,240	48,371	68,409	36,050	0.53
tree5-line5.ply	23,684,400	77,095	144,182	35,567	0.55
tree6-line4.ply	12,180,160	91,759	116,623	32,973	0.55
tree6-line5.ply	23,831,280	122,731	201,853	40,105	0.53
tree6-line6.ply	22,129,920	131,724	156,657	38,092	0.53
tree6-line7.ply	20,844,720	112,263	143,099	18,181	0.49
tree7-line5.ply	11,293,440	36,503	61,915	27,186	0.57
tree7-line6.ply	23,060,160	113,748	154,022	30,720	0.56
tree8-line6.ply	23,011,200	74,667	108,985	31,835	0.56
tree8-line7.ply	24,406,560	131,787	129,985	34,799	0.55
tree8-line8.ply	13,480,320	59,801	137,296	33,443	0.55
tree9-line7.ply	21,591,360	73,581	135,529	37,837	0.52
tree9-line8.ply	26,891,280	78,400	199,679	39,972	0.52

## Limitations

The principal factors affecting the spatial fidelity of the point clouds in this MTLS protocol are the kinematic velocity of the operator and the radial deviations during the orbital trajectory around each pecan tree. Although a physical radial tether was used to reduce this variability, the topographical irregularities of the orchard floor introduced unavoidable fluctuations in position. This geometric instability was further compounded by the vertical oscillations of the operator when capturing the full extent of the woody crown. The low-cost solid-state LiDAR system integrated into the device is sensitive to these biomechanical movements. As a result, kinematic inconsistencies may have exacerbated the SLAM drift, leading to spurious laser returns and increased noise levels in the dataset [13].

## Ethics Statement

The authors confirm that they have read and adhered to the ethical requirements for publication in *Data in Brief*. This work does not involve human subjects, animal experiments, or data collected from social media platforms.

## CRediT authorship contribution statement

**Raúl Porras:** Investigation, Data curation, Writing – original draft. **Gilberto Rivera:** Supervision, Writing – review & editing. **Vicente García:** Project administration, Visualization. **Rogelio Florencia:** Methodology, Resources. **Patricia Sánchez-Solís:** Formal analysis, Conceptualization.

## Data Availability

ZenodoPoint cloud dataset of mature pecan trees (Carya illinoinensis) obtained with a low-cost mobile terrestrial LiDAR (Original data).
